# An Interleukin-17 Isoform from Thick Shell Mussel *Mytilus coruscus* Serves as a Mediator of Inflammatory Response

**DOI:** 10.3390/molecules28041806

**Published:** 2023-02-14

**Authors:** Jiemei Zhao, Zhenyu Dong, Li Zhu, Weihua Song, Pengzhi Qi

**Affiliations:** National Engineering Research Center of Marine Facilities Aquaculture, Marine Science and Technology College, Zhejiang Ocean University, Zhoushan 316004, China

**Keywords:** *Mytilus coruscus*, interleukin-17, RNA interference, flow cytometric analysis, inflammatory response

## Abstract

The inflammatory cytokine interleukin-17 (IL17) plays an important role in innate immunity by binding to its receptors (IL17Rs) to activate immune defense signals. To date, information on members of the IL17 family is still very limited in molluscan species. Here, a novel member of the IL17 family was identified and characterized from thick shell mussel *Mytilus coruscus*, and this gene was designated as *Mc*IL17-1 by predicting structural domains and phylogenetic analysis. *Mc*IL17-1 transcripts existed in all examined tissues with high expression levels in gills, hemocytes and digestive glands. After the stimuli of different pathogen associated molecular patterns (PAMPs) for 72 h, transcriptional expression of *Mc*IL17-1 was significantly upregulated, except for poly I:C stimulation. Cytoplasm localization of *Mc*IL17-1 was shown in HEK293T cells by fluorescence microscopy. Further, in vivo and in vitro assays were performed to evaluate the potential function of *Mc*IL17-1 played in immune response. *Mc*IL17-1 was either knocked down or overexpressed in vivo through RNA inference (RNAi) and recombinant protein injection, respectively. With the infection of living *Vibrio alginolyticus*, a high mortality rate was exhibited in the *Mc*IL17-1 overexpressed group compared to the control group, while a lower mortality rate was observed in the *Mc*IL17-1 knocked down group than control group. In vitro, the flow cytometric analysis showed that the apoptosis rate of *Mc*IL17-1 inhibited hemocytes was significantly lower than that of the control group after lipopolysaccharide stimulation. These results collectively suggested that the newly identified IL17 isoform is involved in the inflammatory response to bacterial infection in *M. coruscus*.

## 1. Introduction

Due to the lack of antibody-based adaptive immunity, bivalves are protected from pathogens by anti-infective effectors such as antimicrobial peptides and a range of more sophisticated defense mechanisms known as innate immunity [1]. Innate immunity represents an ancient evolutionary defense strategy shared by vertebrates and invertebrates for nonspecific recognition of pathogenic microorganisms [2]. Mussels, the largest phyla of bivalves, are frequently exposed to a wide range of microorganisms due to their filtering activity [3]. They rely entirely on the innate immune system to confront pathogen invasion, which is activated by pattern recognition receptors (PRRs) that recognize pathogen-related molecular patterns (PAMPs) or damage-related molecular patterns (DAMPs) [4,5,6].

As one of the oldest PRRs, the toll-like receptors (TLRs) have a broad pattern recognition spectrum and can recognize diverse PAMPs [7], including lipids, lipoproteins, proteins, and nucleic acids derived from a wide range of microbes such as bacteria, viruses, fungi and protozoa [8,9,10,11]. Upon recognition of PAMPs, TLRs drive downstream signaling pathways mediated by the transcription factor NF-κB and IRFs, which in turn triggers the expression of multiple pro- and anti-inflammatory genes, among which interleukin-17 (IL17) is one of the most typical pro-inflammatory factors [12,13].

IL17 is one member of the most ancient cytokine family; six IL17 family ligands (IL17A-F), as well as five receptors of them (IL17RA-E), have been identified in mammals [14]. Structurally, these cytokines generally embrace an IL17 domain at the C-terminus, where four conserved cysteine residues form intra-chain disulfide bonds to promote dimerization [15]. The unique structural characteristics of 4-cysteine enable IL17 to fulfill its function by binding or interacting with its specific receptors, meanwhile providing a basis that distinguishes IL17 sequences from other cytokines [16]. Unlike traditional inflammatory cytokines, activated T lymphocytes and other cell types relevant to the host immunity, such as the neutrophils and the mucosal epithelial cells, produce IL-17s [17,18]. Nevertheless, IL17 plays a fundamental role in innate immunity as its secretion triggers the production of a large number of chemokines, leading to the recruitment of neutrophils and macrophages, which, in turn, clear pathogens [19]. By this means, IL17 mediates the interaction between the innate and adaptive immunity, thereby coordinating an effective immune response [20].

IL17 and its mediated signaling pathway have long been thought to be unique to vertebrates. However, thirty IL17s and two IL17Rs were identified in the genome of sea urchins in 2006, thereby opening the prologue to IL17s in invertebrates [21]. According to written records, the molluscan IL17 was first found in *Crassostrea gigas* in 2008, and two clones encoding a protein analogous to vertebrate IL17s were obtained from *C. gigas* hemocyte cDNA library [22]. Further research showed that the transcriptional expression of *Cg*IL17s was distinctly increased in hemocytes in response to PAMPs, suggesting that *Cg*IL17s can participate in innate immune response [23]. Moreover, an IL17 receptor named *Cg*IL17R1 was also identified from *C. gigas*, which binds to *Cg*IL17 in granulocytes to mediate hemocyte proliferation during immune modulation of *C. gigas* [24]. This may shed some new light on the regulation of hemocyte proliferation in invertebrates. In *Pinctada fucata*, IL17 has proven to be capable of activating the NF-κB signal pathway against exogenous pathogens [25]. Several IL17s and -17Rs have also been identified in other mollusks, including *Haliotis discus hannai* and *Mytilus galloprovincialis* [26,27,28,29], which provided valuable clues to elucidate the functional roles of IL17s in innate immune regulation in mollusks.

The thick shell mussel *Mytilus coruscus*, a species of mussel in the Mollusca genus Bivalvia, is primarily found in the western Pacific waters [30]. *M. coruscus* has evolved over the past several years into a model organism for investigating the physiological response and immune reactivity of molluscans to environmental stress because of its enormous economic relevance and ubiquity in coastal waters [31,32]. The clarification of the underlying mechanism for innate immune response is of great academic significance. Here, we molecularly identified an IL17 homolog (*Mc*IL17-1) from *M. coruscus*. Following its transcriptional response to multiple PAMPs, *Mc*IL17-1 effects on mussel survival and hemocytes apoptotic rate were assessed through laboratory means. The present study is helpful in filling the theoretical gap of the IL17 in *M. coruscus*, while providing some additional information to elucidate the role of proinflammatory factors acting in mollusks.

## 2. Results

### 2.1. Molecular Cloning and Characterization of McIL17-1

**Figure 1:**
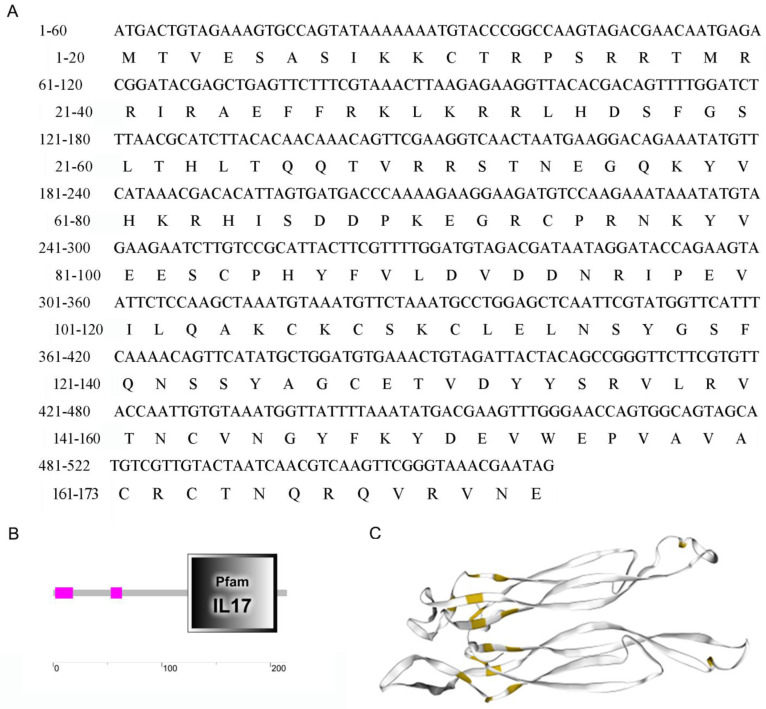
Molecular characterization of *Mc*IL17-1. (**A**) The nucleotide sequences and the deduced amino acid sequences of IL17-1 in thick shell mussel *Mytilus coruscus*. It contains a 585 bp ORF region coding the protein of 194 amino acid residues; (**B**) Schematic diagram of IL17 structure domain; (**C**) Schematic diagram of cysteine nodule structure (Yellow marked: cysteines).

**Figure 2:**
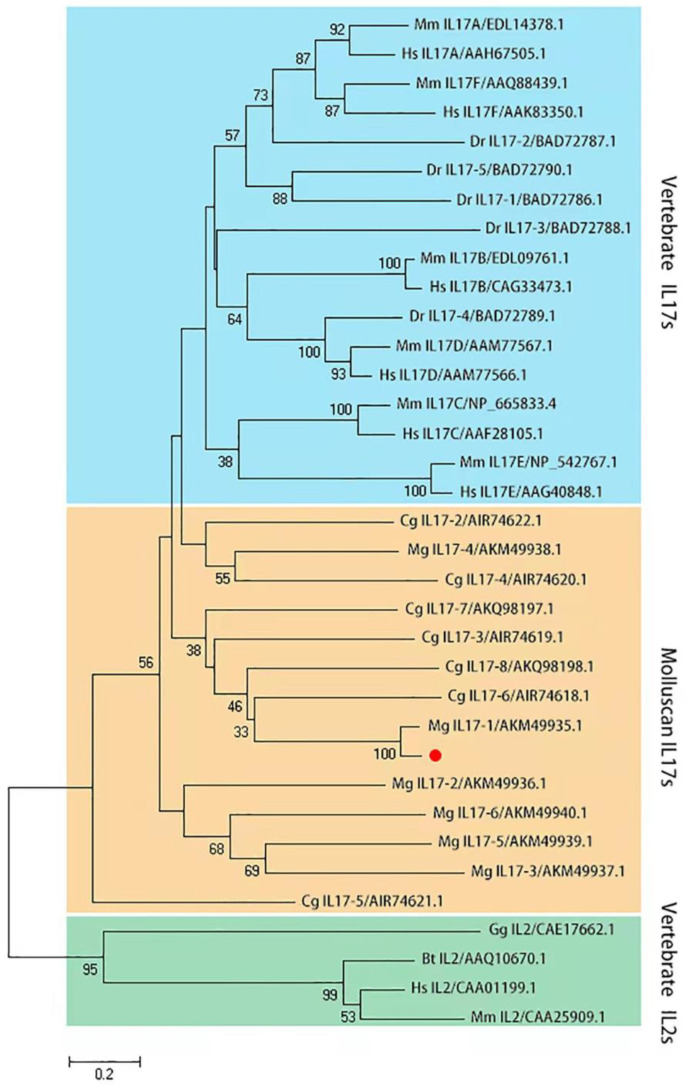
Phylogenetic analysis of *Mc*IL17-1. The phylogenetic tree is constructed using MEGA-X software with 2000 replications of bootstrap in the way of the neighbor-joining method. *Mc*IL17-1 is labeled with a red dot. Species included in the phylogenetic tree are all retrieved from the GenBank database and accession numbers are also listed in the tree. Sequences from various species are abbreviated as follows: *Hs*, *Homo sapiens*; *Mm*, *Mus musculus*; *Bt*, *Bos taurus*; *Dr*, *Danio rerio*; *Gg*, *Gallus gallus*; *Mg*, *Mytilus galloprovincialis*; *Cg*, *Crassostrea gigas*.

The *Mc*IL17-1 cDNA containing the complete ORF region was cloned from *M. coruscus* (GenBank: GCA_011752425.2). *Mc*IL17-1 contains a 585 bp ORF region encoding 194 amino acids (Figure 1A). The predicted molecular weight is 20.35 kDa, and the isoelectric point is 9.43. SMART analysis revealed a typical IL17 domain in this protein (Figure 1B). A phylogenetic tree was constructed with vertebrate interleukin-2 cytokines (IL-2s) as outgroup. Clearly, these IL17s clustered into a distinct clade to distinguish them from IL-2s. In the IL17s cluster, *Mc*IL17-1 first bound to its corresponding molecule in another *Mytilus* species i.e., *Mytilus galloprovincialis*, and then clustered with other IL17s from *M. galloprovincialis* and *Crassostrea gigas* into one subbranch. In addition, the vertebrate IL17s converged into a distinct clade distinct from the molluscan IL17s clade (Figure 2).

### 2.2. Transcriptional Expression of McIL17-1

**Figure 3:**
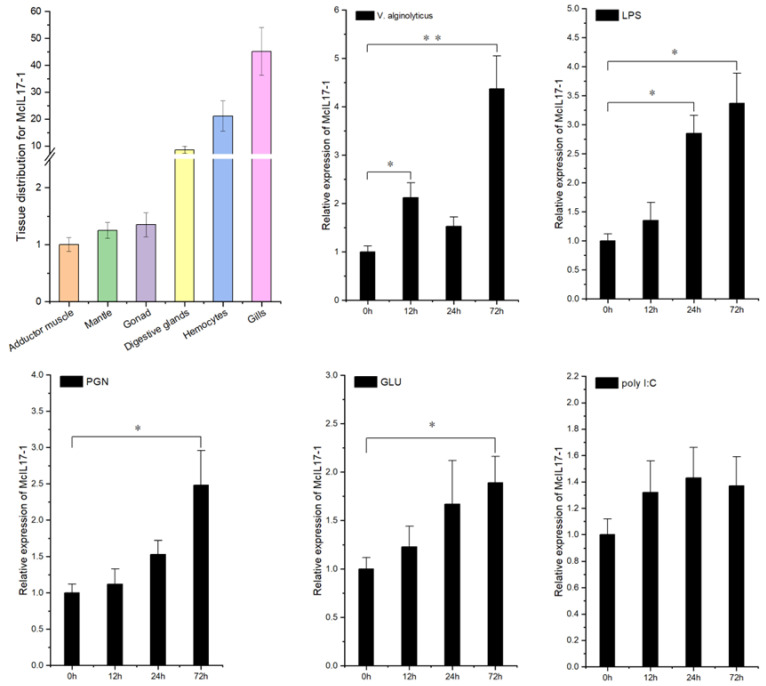
Expression profile analysis of *Mc*IL17-1 transcripts in common adult tissues and after challenge with live *V. alginolyticus*, lipopolysaccharide (LPS) (from *Escherichia coli* O111: B4), peptidoglycans (PGN) (from *Staphylococcus aureus*), glucan (GLU) (from *Saccharomyces cerevisiae*), or polyinosinic-polycytidylic acid (poly I:C), respectively. The results were expressed as mean ± S.D. (n = 3). Significant difference relative to control is indicated with asterisk symbol (* *p* < 0.05, ** *p* < 0.01).

The tissue distribution profile of *Mc*IL17-1 transcripts was assessed by qPCR assay. As shown in Figure 3A, the expression level of *Mc*IL17-1 mRNA was the highest in gills, followed by in hemocytes and digestive glands, and lowest in adductor muscle. Additionally, the transcriptional response to multiple PAMPs was also evaluated for *Mc*IL17-1 (Figure 3B). With the stimulation of live *V. alginolyticus*, the transcriptional expression of *Mc*IL17-1 was significantly upregulated at 12 and 72 hps. Similarly, *Mc*IL17-1 transcripts also showed a response to other immune stimuli, such as LPS, PGN and GLU, which all peaked at 72 hps. However, poly I:C injection did not significantly change the mRNA level of *Mc*IL17-1.

### 2.3. Subcellular Localization of McIL17-1

**Figure 4:**
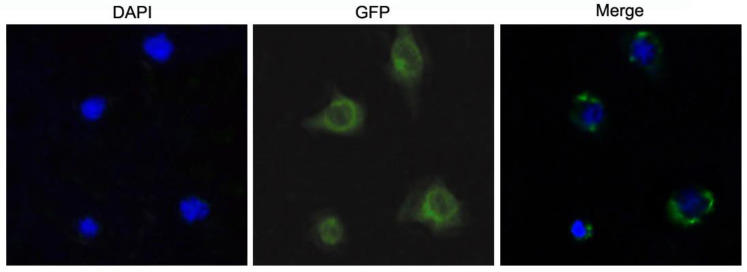
Subcellular localization of *Mc*IL17-1 in HEK293T cells. The recombinant pEGFP-N1-*Mc*IL17-1 plasmid was transfected into HEK293T cell using lipofectamine 3000, the green fluorescence showed the location of proteins and the cell nucleus location was indicated by blue DAPI staining. The *Mc*IL17-1 was mainly localized in the cytoplasm of HEK293T cells.

Due to the absence of mature mussel cell lines, the cellular localization of *Mc*IL17-1 was examined in the HEK293T cells through the transfection of the constructed pEGFP-N1-*Mc*IL17-1 plasmid. Under a fluorescence microscope, the green fluorescence of GFP-labeled *Mc*IL17-1 was primarily found in the cytoplasm (Figure 4).

### 2.4. Effects of Reduction and Overexpression of McIL17-1 on Survival Rate of Mussels

**Figure 5:**
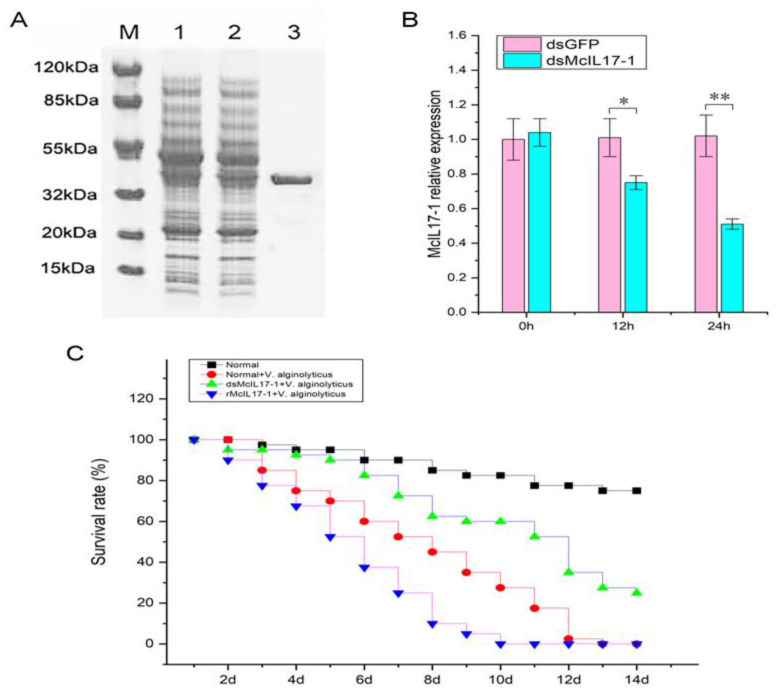
Effects of silencing and overexpression of *Mc*IL17-1 on survival rate of mussels. (**A**) SDS-PAGE analysis of r*Mc*IL17-1. Lane M: protein molecular standard; lane 1: negative control for r*Mc*IL17-1(without induction); lane 2: transfected r*Mc*IL17-1; lane3: purified r*Mc*IL17-1. (**B**) *Mc*IL17-1 was silenced through dsRNA injection. (**C**) The survival rate (%) of mussels prior to ds*Mc*IL17-1 and r*Mc*IL17-1 injection following *V. alginolyticus* challenge. **, *p* < 0.01, *, *p* < 0.05 versus the controls.

*Mc*IL17-1 was recombinantly expressed and purified by the pET-32a prokaryotic expression system. The molecular mass of r*Mc*IL17-1, rTrx, His tag protein and cohesive amino acids combined together was compatible with the band size of roughly 40 kDa that appeared on the SDS-PAGE (Figure 5A). After injection of ds*Mc*IL17-1, the mRNA expression level of *Mc*IL17-1 was significantly decreased in *M. coruscus* hemocytes (0.74 and 0.52 fold at 12 h and 24 h compared to control, respectively) (Figure 5B). Purified r*Mc*IL17-1 was injected into healthy mussels, and a total of four groups of mussels, i.e., normal group, *V. alginolyticus* treated group, *V. alginolyticus* and ds*Mc*IL17-1 co-treated group, *V. alginolyticus* and r*Mc*IL17-1 co-treated group were employed to assess the effects of silencing and overexpression of *Mc*IL17-1 on survival rate of mussels. The results showed that the mussels of *V. alginolyticus* and r*Mc*IL17-1 co-treated groups all died on the 10th day, prior to *V. alginolyticus* solely treated mussels that all died on the 13th day (Figure 5C). After infection with the *V. alginolyticus*, these *Mc*IL17-1 silenced mussels continued to die over time. However, the mortality rate was significantly lower than the other two *V. alginolyticus* treated groups (Figure 5C).

### 2.5. Hemocytes Apoptotic Rate after McIL17-1 Was Inhibited

**Figure 6:**
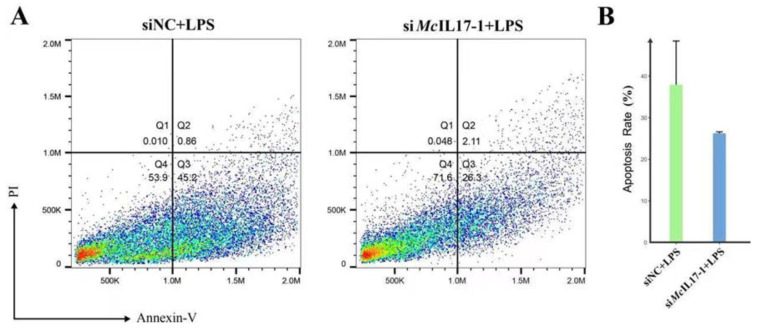
Apoptotic rate in hemocytes of *M. coruscus* after LPS treatment and after *Mc*IL17-1 was knocked-down. The apoptotic rate of hemocytes was detected by flow cytometry with Annexin V-FITC and propidium iodide (PI) staining. (**A**) Apoptotic rate in RNAi mussel hemocytes. siNC + LPS: The apoptosis rate of hemocytes at 24 h after LPS challenge in siNC group; si*Mc*IL17-1 +LPS: The apoptosis rate of hemocytes at 24 h after LPS challenge in si*Mc*IL17-1 group. Quadrant: Q4: live cells; Q3: early apoptotic cells; Q2: late apoptotic cells; Q1: necrotic cells. (**B**) The changes of the apoptotic rate of hemocytes after dsRNA challenge.

The apoptosis rate of hemocytes after LPS treatment was analyzed by flow cytometer after *Mc*IL17-1 was inhibited. For the mussels in siNC group (negative control siRNA), the apoptosis rate of hemocytes was 46.1%. However, the apoptosis rate decreased to 28.4% in si*Mc*IL17-1 group, which was significantly lower than that in siNC group (Figure 6).

## 3. Discussion

Innate immunity is the main defense mechanism against pathogenic infections in marine invertebrates [33]. Since its discovery in human peripheral blood, the soluble proinflammatory cytokine IL17 family, has been widely studied as one of the key signaling molecules of innate immunity [34]. In terms of medical research, IL17 expression has long been related to transplant rejection of various solid organs such as kidney and heart [35]. In addition, IL17 also plays a role in other pathophysiological processes, including host bacterial defense, granulopoiesis, rheumatoid arthritis, tumor regulation, and asthma [36]. Consequently, any dysregulation of IL17 production and disturbance of downstream signal pathways may affect the normal physiology and function of disease pathogenesis in human. In view of its significant functional role in innate immunity, the IL17 gene family has been paid more attention in aquatic animals in the past decade [37,38,39,40,41,42]. Additionally, as genome sequencing and resequencing technologies have advanced by leaps and bounds over the past two decades, members of the IL17 gene family in mollusks have been revealed in growing numbers [24,27,28]. Most of these studies focused on the IL17 evolution and immunological function verification, providing a prelude to elucidate the immune role of IL17 family in shellfish. Nevertheless, the mechanisms underlying IL17 mediated innate immune response to bacterial infection remain unclear. Here, a novel molluscan IL17 family member (*Mc*IL17-1) was identified from *M. coruscus* mussels. Structural analysis of *Mc*IL17-1 showed an IL17 domain, which contains four conserved cysteine. In the phylogenetic tree, *Mc*IL17-1 clustered with *Mg*IL17-1 and aggregated in a molluscan IL17 cluster. Based on the conservation in structural domain with its corresponding genes in other bivalves, we further explored the role of *Mc*IL17-1 in innate immune restriction.

As an essential mediator of inflammatory autoimmune diseases, IL17 is involved in innate immunology responses in many tissues [16]. Pisces represent the first group in fauna evolution to have both innate and adaptive immunity [42]. In zebrafish, tissue distribution patterns of IL17 transcripts showed that *Dr*IL17 mRNA was expressed in the kidney, spleen, gills and intestine [43], which is consistent with previously reported results from turbot (*Scophthalmus maximus*) [41]. Moreover, a number of studies have labelled the constitutive expression of IL17 in molluscan species. For example, in Pacific oyster, IL17 mRNA was detected in all examined tissues, including hemocytes, heart, gills, digestive glands, gonad, mantle and muscle, and highly expressed in the gills and digestive glands tissues [23]. For freshwater pearl mussel *Hyriopsis cumingii*, IL17 was also found in any tissues tested, with the gills and digestive glands having the highest levels of IL17 [29]. In the present study, *Mc*IL17-1 also showed a broad spectrum of expression and significantly higher expressed in gills and hemocytes compared with that in other tissues. In mollusks, gills mediate mucosal immunity and are generally regarded as the first line of defense against infection by various pathogens [44]; hemocytes are thought to be essential immunological agents that are critical for cellular and humoral immunity in the fight against pathogen invasion [6]. Considering that *Mc*IL17-1 is highly expressed in hemocytes and gills, it is feasible to function similarly to its counterparts in other mollusks in the innate immune response.

As a potent proinflammatory cytokine, the production of IL17 in mammals is induced by infection and is thought to drive tissue inflammation and autoimmune disease [45]. To further determine the functional role of *Mc*IL17-1 in innate immunity, we investigated its transcriptional response to the challenge of *V. alginolyticus*, a main pathogenic bacteria of bivalve mollusks [46,47]. Following stimulation by *V. alginolyticus*, the mRNA expression of *Mc*IL17-1 in hemocytes increased promptly, indicating an effective response to bacterial infection. Similar results have also been observed in *Crassostrea gigas* and *Mytilus galloprovincialis*. For *C. gigas*, the mRNA expression of *Cg*IL17-1 in the hemocytes increased significantly at 6 h post-*V. splendidus* challenge, which was 31.36-fold of that in the control group [24]. The *Mg*IL17-1 transcripts in hemocytes also showed significant upregulation after *M. galloprovincialis* was injected with a mixture of heat-killed Gram+ and Gram- bacteria [28]. Sensing PAMPs is a crucial step in innate immune activation [48]. Aiming to investigate whether *Mc*IL17-1 participates in innate immune response to various exogenous pathogens, we characterized the transcriptional expression of *Mc*IL17-1 in response to four PAMPs, including LPS, PGN, GLU and poly I:C. Concomitantly, *Mc*IL17-1 was significantly induced by LPS, PGN and GLU, similar to the results of upregulated transcript levels of *Cg*IL17s in response to PAMPs challenge in the oysters [23]. An intriguing aspect was that the *Mc*IL17-1 expression of poly I:C-injected mussels did not change significantly compared to the control group, which was different from the parallel study result of *P. fucata* [25]. In *P. fucata*, *Pf*IL17 mRNA expression was upregulated significantly by poly I:C. In contrast, for *C. gigas*, poly I:C challenge raised the mRNA level of almost all *Cg*IL17s, except *Cg*IL17-1, indicating that *Cg*IL17s were generally involved in fighting viral infection, but *Cg*IL17-1 may lose this role [23]. For vertebrates, LPS and poly I:C did not affect IL17N expression in Atlantic salmon head kidney cells [49]. The responses to bacteria and viruses are generally different, which is reflected in the different IL17 responses. Considering that LPS and poly I:C are substitutes for bacteria and viruses, respectively, the susceptibility to LPS and the bluntness to poly I:C suggested that *Mc*IL17-1 may be involved in the inflammatory response to bacterial infection. In addition, the responsive reactions of *Mc*IL17-1 to various of exogenous stimuli also indicated that *Mc*IL17-1 has a broad spectrum of action to include bacteria and fungi and less for viruses.

As a cytokine associated with inflammation and autoimmunity, IL17 induces the expression of various mediators of inflammation [20]. In vertebrates, most experimental evidence suggests that members of the IL17 family play a role in coordinating local tissue inflammation, primarily through the release of proinflammatory and neutrophil-mobilizing cytokines [50]. For example, IL17s in mice have been reported to play an important role in host defense against bacterial infections by inducing CXC chemokines to recruit neutrophils and induce antimicrobial proteins at the site of infection [51]. In human, IL17A and several other family cytokines are also involved in the development of psoriatic arthritis, psoriasis and ankylosing spondylitis by inducing inflammatory cytokines and chemokines [52]. For bivalves, a series of inflammatory reactions caused by bacterial infection is the leading cause of death, especially *Vibrio* sp. bacteria [46]. In the present study, we further explored the possible role of *Mc*IL17-1 as an inflammatory cytokine in mediating the pathogenesis of bacterial infections by evaluating the effects of silencing and overexpression of *Mc*IL17-1 on mussel survival. The present results showed that the overexpression of *Mc*IL17-1 elevated the mortality rate. In contrast, the survival rate of the knocked-down group was significantly higher than that of *V. alginolyticus* alone treated group and overexpression group. These results revealed that suppression of *Mc*IL17-1 expression reduced the inflammatory response and increase the survival rate, which further reinforced the point that *Mc*IL17-1 was involved in disease promoting proinflammatory processes in response to bacterial infection.

The analysis of the subcellular localization of a protein can provide insight to its function. We performed cytoplasmic localization of *Mc*IL17-1 by fluorescence microscopy in the HEK293T cell system. The results showed that *Mc*IL17-1 was located in the cytoplasm, which was in agreement with previous studies that the *Cg*IL17-1 and IL17s of *Scophthalmus maximus* were subcellular located in the cytoplasm of hemocytes from *C. gigas* and HEK293T cells, respectively [24,41]. IL17 functions via binding to its cognate receptor and more research is needed to confirm the binding site [20].

As a form of programmed cell death, apoptosis plays a role in both the development of immune cells and the execution of an immune response. Meanwhile, apoptosis is also an important mechanism for maintaining immune homeostasis [53]. In mice, IL17 has been proven to induce endothelial apoptosis by inducing caspase-9, caspase-3 and raising the ratio of Bax/Bcl-2 [54]. However, there has not yet been one literature report that IL17 regulates apoptosis in invertebrate cells. LPS, as a cell-wall component of gram-negative bacteria and has been increasingly recognized as a powerful stimulator of cellular immunity in previous studies [55]. In oysters, it has been reported that the apoptosis of hemocytes significantly increased after LPS treatment [56], following *Cg*Smac was employed to activate the mitochondrial apoptosis pathway by enhancing caspase-3 activity to resist exogenous LPS invasion [57]. In the present study, the effect of *Mc*IL17-1 on hemocytes apoptosis was assessed using LPS as the inducer. The apoptosis rate of the si*Mc*IL17-1 group decreased significantly compared with the siNC group, which suggested that *Mc*IL17-1 could promote apoptosis of hemocytes challenged by LPS in *M. coruscus*. Given that apoptosis plays a crucial role in pushing the resolution of acute inflammatory responses, the present results suggest that *Mc*IL17-1, a proinflammatory cytokine, mediates inflammation and is produced in response to challenge [58]. Therefore, the normal levels of *Mc*IL17-1 expression contribute to the pathogenesis of bacterial infections.

## 4. Materials and Methods

### 4.1. Animals

Healthy adult *M. coruscus* (about 8.0–10.5 cm in shell height) with an average weight of 60.4 ± 3.2 g were obtained from Donghe market in Zhoushan City, Zhejiang Province, China. The domestication conditions were consistent with previous studies [59]. Briefly, animals were kept in tanks with artificial sterile seawater (ASW) at 25 ± 1 °C, salinity 25%, and fed with spirulina powder.

### 4.2. In Silico Cloning for McIL17-1

The sequence information of *Mc*IL17-1 (GenBank: GCA_011752425.2) was obtained from the National Center for Biotechnology Information (http://www.ncbi.nlm.nih.gov/). A specific primer pair (Table 1) was designed to amplify the sequence of the *Mc*IL17-1 open reading frame (ORF) region. After DNA sequencing of the PCR products, the physicochemical characteristics of *Mc*IL17-1 were assessed by a website tool Expasy (http://www.expasy.org) and SMART (http://smart.embl-heidelberg.de/) was used to predict the conservative domains. The phylogenetic tree was constructed by the MEGA X software package with Neighbor-joining (NJ) method, and bootstrap resampling (2000 pseudo repetitions) was conducted to test the reliability of branches.

### 4.3. Immune Challenge and Tissue Collection

A total of 250 mussels were randomly divided into five groups. The immune challenge assay was performed as in our previous study [60], in a word, the mussels of five treatment groups were injected in the adductor with 100 μL live *Vibrio alginolyticus* (3 × 10^7^ CFU/mL), lipopolysaccharide (LPS) (L3024, Sigma, from *Escherichia coli* O111: B4, 50 μg/mL), peptidoglycans (PGN) (77140, Sigma, from *Staphylococcus aureus*, 0.5 mg/mL), glucan (GLU) (G5011, Sigma, from *Saccharomyces cerevisiae*, 1 mg/mL), or polyinosinic-polycytidylic acid (poly I:C), (P9582, Sigma, 1 mg/mL), respectively. Of these, *V. alginolyticus* was obtained from the diseased mussels as the pathogenic bacteria and the rest were purchased from Sigma-Aldrich (Shanghai). Samples of hemolymph were collected from the pericardium of mussels at 0, 12, 24 and 72 h post-stimulation (hps). Hemocytes were obtained by centrifugation at 1100× *g* rpm for 10 min at 4 °C. There were 3 replicates for each time point, and the hemocyte samples from 3 mussels were pooled together as one replicate to alleviate the individual variation and obtain enough cells. Each time point consisted of three replicates. Moreover, six tissues, including adductor muscle, gills, mantle, gonad, hemocytes and digestive glands, were extracted from eight untreated mussels to analyze the tissue distribution of *Mc*IL17-1.

### 4.4. Expression and Purification of Recombinant McIL17-1 Protein

One specific primer pair IL17-1Y1 (Table 1), incorporated with BamH I and XhoI restriction sites at its 5′ end was designed to amplify the full length of the *Mc*IL17-1 ORF sequence. A recombinant plasmid termed pET-32a-*Mc*IL17-1 was generated by subcloning the *Mc*IL17-1 ORF sequence into the pET-32a prokaryotic expression vector, following transformation into *Escherichia coli* cells (DE3) (Takara) to express the fusion proteins. The induction and purification of the recombinant protein (r*Mc*IL17-1) were performed according to our previous report [61], that the isopropyl-beta-D-thiogalactopy ranoside (IPTG) and Ni-nitorilotriacetic acid (NI-NTA) used as the inducer and depurator, respectively. The purified r*Mc*IL17-1 was analyzed using SDS-polyacrylamide gel electrophoresis (SDS-PAGE) and visualized with Coomassie brilliant blue R250. The concentration of purified recombinant protein was quantified by the BCA method [62].

### 4.5. RNA Interference

As we described earlier, the RNA interfere assay (RNAi) was performed through the dsRNA injection [61]. The dsRNA was synthesized by T7 polymerase using *Mc*IL17-1 cDNA sequence amplified by specific primer pair (Table 1, IL17-ds) as the template. Following that, the obtained substance was transferred into the mussel by adductor injection (100 μg per mussel), and another booster shot was given 24 h later. After receiving a second dsRNA injection, the mussels were given treatment with *V. alginolyticus* 12 h later. During the experiment, a total of 40 mussels from each group were fed with spirulina powder and seawater was changed 2 h after feeding. Meanwhile, the number of mussel deaths was recorded daily [63].

### 4.6. qPCR

Quantitative real-time PCR (qPCR) was performed on the 7500 Real-Time PCR system (Applied Biosystems, Foster City, CA, USA) with the SYBRR^®^ premix ExTaq Kit (TaKaRa, Kusatsu, Japan) to assess the transcriptional expression of *Mc*IL17-1, as previously reported [61]. The qPCR reaction system was conventional, and the reaction conditions were as follows: 95 °C pre-denaturation for 10 min, 40 cycles of 95 °C denaturation for 10 s and 58 °C annealing for 20 s. Data were analyzed using the 2^–ΔΔCT^ method with β-actin as an internal reference [64]. Three analyses were performed on all samples.

### 4.7. Subcellular Localization

The *Mc*IL17-1 fragment was cloned into the pEGFP-N1 vector with one specific primer pair IL17-1Y2 (Table 1) incorporated EcoR I and BamHⅠrestriction sites, followed by a DNA sequencing verification. Due to no real established cell lines available for marine bivalves, HEK293T cells were used. HEK293T cells were inoculated in sterile 6-well plates at 60–70% density. After cell adhesion was complete, the transfection reagent Lipofectamine 3000 (Invitrogen, Waltham, CA, USA) and recombinant plasmid pEGFP-N1-*Mc*IL17-1 were mixed 1:1 to form the transfection complex. The transfection complex was repeatedly blown 10 to 15 times with a pipette, then transferred to a 6-well plate, mixed gently, and placed in a cell incubator. After 48 h of culture, 4% paraformaldehyde was used for fixation. 4′,6-diamidino-2-phenylindole (DAPI, 1 μg/mL) was used for nuclear staining after PBS cleaning. The position of *Mc*IL17-1 in cells was observed by fluorescence microscopy.

### 4.8. Flow Cytometric Analysis of Apoptosis

Hemocytes apoptosis was assessed and quantified according to the manual of Annexin V-FITC Apoptosis Detection Kit (Beyotime, PK, Nantong, China). Briefly, the collected hemocytes were treated with LPS (0.1 mg/mL), and either si*Mc*IL17-1 (10 μL, 2 μM) or si-NC for 24 h. After washing with PBS, the cells were re-suspended in the L15 medium at a final concentration of 1 × 10^6^ cells mL^−1^, and then they were stained with Annexin V-FITC and PI by being incubated at room temperature for 25 min in the dark. Finally, the flow cytometry instrument (Beckman CytoFLEX FCM) was employed to detect cell apoptosis, and data were analyzed using FlowJo software (New York, NY, USA).

## 5. Conclusions

In this work, a novel IL17 isoform was identified and characterized from the thick shell mussel *Mytilus coruscus*. The increased expression in immune-related tissues and effective responsiveness to PAMPs suggest that it is involved in the innate immune response. In vivo and in vitro assays further reinforce the idea that *Mc*IL17-1 may function as a proinflammatory cytokine in the immune response to bacterial infections. The present study hints at the complexity of invertebrate immunity.

## Figures and Tables

**Table 1:** PCR primer pairs used in the present study.

Primer	Sequences (5′–3′)	Usage
IL17-1	ATGGTTATTTTGATACTGCAAAC	For *Mc*IL17-1 ORF cloning
	TCAATTAATTCGATTTTCAATT	
IL17-1Y1	CACGGATCCATGGTTATTTTGATACTGCAAAC	For pET-32a-*Mc*IL17-1 plasmid construction
	GACCTCGAGTCAATTAATTCGATTTTCAATT	
IL17-1Y2	CAGAATTCATGGTTATTTTGATACTGCAAAC	For pEGFP-N1-*Mc*IL17-1 plasmid construction
	GAGGATCCATTAATTCGATTTTCAATT	
IL17-1-ds	TAATACGACTCACTATAGGGATGGTTATTTTGATACTGCAAAC	For *Mc*IL17-1 gene silencing
	TAATACGACTCACTATAGGGATTAATTCGATTTTCAATT	
GFP-ds	TAATACGACTCACTATAGGGATGGTGAGCAAGGGCGAGGA	Negative control in RNAi
	TAATACGACTCACTATAGGGTTACTTGTACAGCTCGTCCA	
Real-IL17-1	TGTGGTTATGAGGGCAACGA	For *Mc*IL17-1 qPCR
	TGGTCGTTTACAAGCACATCC	
β-actin	GCTACGAATTACCTGACGGACAG	Internal reference
	TTCCCAAGAAAGATGGTTGTAACAT	

## Data Availability

Not applicable.
